# Lapses of the Heart: Frequency and Subjective Salience of Impressions Reported by Patients after Cardiac Arrest

**DOI:** 10.3390/jcm12051968

**Published:** 2023-03-02

**Authors:** Fritz Sterz, Michael L. Berger, Gerhard Ruzicka, Roland Beisteiner

**Affiliations:** 1 Department of Emergency Medicine, Medical University of Vienna, Währinger Gürtel 18-20, A-1090 Vienna, Austria; 2 Center for Brain Research, Medical University of Vienna, Spitalgasse 4, A-1090 Vienna, Austria; 3 Department of Neurology, Medical University of Vienna, Währinger Gürtel 18-20, A-1090 Vienna, Austria

**Keywords:** cardiac arrest, cardiopulmonary resuscitation, structured interview, subjective experience, symbolic meaning, meeting deceased relatives

## Abstract

After cardiac arrest (CA), some patients report impressions with highly realistic features, often referred to as near-death experience (NDE). The frequency of such episodes seems to be variable, with various types of content. In a prospective study, we subjected 126 CA cases treated at the Department of Emergency Medicine of the Medical University of Vienna under carefully controlled conditions to a structured interview. We included all patients admitted due to CA, whose communicative abilities were restored and who agreed to participate in the study. The questionnaire inquired as to living conditions, attitudes towards issues of life and death, and last recollections before and first impressions after the CA. The majority of the subjects (91 = 76%) replied to inquiries concerning impressions during CA with “nothing” or “blackout”, but 20 (16%) gave a detailed account. A German version of the Greyson questionnaire specifically addressing NDE phenomena (included towards the end of the interview) resulted in ≥7 points in five patients (4%). Three patients reported a meeting with deceased relatives (one with 6 Greyson points), one an out-of-body episode, and one having been sucked into a colorful tunnel. Eleven of these twenty cases had their cardiopulmonary resuscitation (CPR) started within the first min of CA, a higher fraction than cases without experience. Reported experience after CA was of high significance for the patients; many of them changed their point of view on issues of life and death.

## 1. Introduction

Considerable periods of time without spontaneous heart activity can be bridged by persistent cardiac massage until professional help sets in. Even if only a small fraction of normal cerebral blood flow is maintained during this procedure, it may be sufficient to delay the irreversible loss of function [1,2]. In general, once brought back to consciousness, such patients do not recall anything of this acute period. Nevertheless, a few report impressions that a skeptical observer from outside would consider improbable [3]. In rodent experiments [4] and in a recent single case human study [5], coherence in all EEG frequency bands was demonstrated to persist for a short time after the arrest of circulation, opening a time window for sustained neuronal activity after cardiac arrest (CA). During the immediate and early periods after CA, brain areas of no flow, low flow, and increased flow may coexist for some time [6], allowing transient and localized neuronal activity. After recovery, a few resuscitated patients report impressions, sometimes including characteristic features termed *near-death experiences* (NDE; [7]) or, more recently, *recalled experiences of death* [8].

One highly popularized account allows the patients—without enough energy in the brain to maintain vital functions—a view of themselves, mostly from an elevated point (*out-of-body experience*; [9]). To put such claims to the test, images were placed only perceptible from above (see, e.g., the AWARE study [10]). During our participation in this multicenter project, we introduced a technical improvement at the Vienna arm. Instead of physical images mounted manually, we used a notebook PC displaying images selected at random from a large pool. This modification strongly reduced the likelihood that knowledge of these images would, by any channel, diffuse to the public. It may be objected that an experimental approach testing for visual awareness from a point outside the body was futile and misplaced in a serious scientific study, neglecting the generally accepted view that ‘even the most complex psychological processes derive from operations in the brain’ [11]. On the other hand, our certainty about the biological basis of awareness (as about any scientific ‘fact’) is the result of well-controlled experiments and observation, but can never be final and absolute. It has always been the noble privilege of experimental research to put to the test even the most solid dogma, provided the chosen approach was sufficiently well controlled against error and fraud.

Our main motivation to embark into this study was the prospect to encounter unusual cognitive phenomena, triggered by the transient cessation of energy supply to the human brain. We expected a low frequency of such events and that, in case they occurred, they could be traced to natural origins. One year and 23 interviews later, we had not seen a single interesting case. During the next 7 years of the project, we were rewarded for our patience with several impressive reports. At the end, we even had reason to examine the presentation history of the hidden image generator.

## 2. Methods

### 2.1. Project Design

The study started as participation in the multicenter project AWARE [10]. After the acquisition of funding, we continued for 7 more years on our own terms. Patient contacts were sustained in the background of the busy routine of a large general hospital. The single questionnaire was informative but short; it could be completed in 20–30 min. Care was taken not to interfere with the treatment of the patients. We agreed to check the hidden image presentation history only after the end of the study.

### 2.2. Patients

Patients were acute admissions to the Department of Emergency Medicine for heart problems. The majority of these problems consisted in CA suffered at home (35.7%), in a hospital (13.5%), on the street (10.3%), or during sport (8.7%). Out-of-hospital CAs were followed by at least one further CA at the emergency unit. Most patients either suffered their CA in their later professional life or a few years after retirement (mean age 58 ± 13 years). The restoration of spontaneous cardiac activity was often followed by physical cooling to 34–32 °C for 12–24 h [12]. The key importance of the interviews limited us to cases with mental and linguistic capability sufficiently restored to allow a meaningful dialogue. Inclusion criteria were (1) admission to the Department of Emergency Medicine and (2) loss of consciousness due to the cessation of blood supply to the brain after CA. Exclusion criteria were (1) incapability to respond to the interview questions and (2) decline of the interview. Among the 126 patients finishing the interviews were 35 women (28%). Consecutive numbers were assigned to the patients in the chronological succession of their CAs. Key data of all 126 cases are listed in Supplementary Material File S1.

### 2.3. Hidden Images

At an elevated position above one emergency bed (2 m above ground), a notebook PC was fixed facing the ceiling and displaying images selected at random from a pool of 29, switching from the actual to any in the pool every few hours (the number of hours was unpredictable). These images were not disclosed to the public and were not even known to all of us (in particular not to the main interviewer M.L.B.). The presentation history was stored on the PC, and any readout of this history, be it authorized or not, left its trace. Only one case gave us reason to examine the image in question. Readout was effectuated after closing the study in 2021; no other readout was noticed.

### 2.4. Interviews

We adhered to the questionnaire of the preceding study [10] (with minor adaptations) in German language (see Supplementary Material File S2). It consisted of 5 parts: (A) General data. (B) Living conditions, questions concerning religion. (C) Last memories before CA, first impressions after regaining consciousness, anything in between, dreams. (D) The 16 Greyson questions. (E) Similar experiences in the past? Of relatives or friends? Known from the media? Cognitive problems since then? Thus, questions concerning the acute interval were not only embedded in other inquiries avoiding exaggerated focus, but were also accompanied by gathering supportive information helpful in judging the relevance of the answers. In dealing with matters of life and death, information about religious background appears relevant. We approached the patients open-minded and unbiased, without insinuating any expectations from our side.

**Figure 1:**
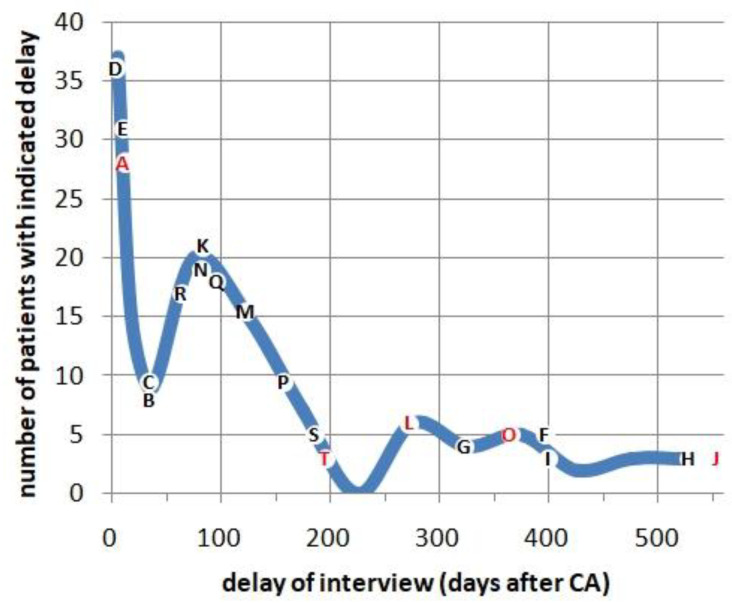
Frequency distribution of interview delay. Interviews were conducted a few days up to 588 days after the CA. The thick line represents all 126 cases. The delay was short for 61 (48.4%), middle for 42 (1/3), and long for 23 cases (18.3%). Cases with detailed recollections (capitals A–T) were slightly more often associated with long delays (χ^2^ 6.66, 0.025 < *p* < 0.05). Capitals in red: ≥7 Greyson points.

Interviews were conducted either during recovery in hospital, or at a later point in time (often at home). The fraction of patients with recollections (see next paragraph) seemed to increase slightly with the time elapsed between CA and interview (Figure 1). If possible, this delay was kept short, but several cases required long-term recovery. *Personal construction* (as e.g., proposed by George A. Kelly [13]) may have influenced their account. We encouraged the presence of a close family member during the interview. This relative was helpful in elucidating the circumstances of the CA (and was often the person that had provided first aid). A short version limited to the central section (C) was sent to 40 patients by mail, who could not be reached by phone, with the suggestion to contact us in case they were interested in participating. Nine of these accepted the invitation, and two (№ 87 and 103) yielded a detailed account of impressions after their CA.

### 2.5. Patients with Recollections

A widely used instrument to investigate subjective experience in resuscitated patients is the questionnaire developed by Bruce Greyson in 1983 [7] on the basis of 67 testimonies solicited from members of the International Association for Near-Death Studies, ‘who believed they had had NDEs as described in the phenomenological literature […] rather than unselected individuals who had come close to death, in order to increase the number of positive responses to the questionnaire’. In contrast, the patients in our study had not been included on the basis of any subjective impressions but only because they had survived a CA with sufficient mental recovery to answer questions. Only 5 of 126 (4%) scored at least 7 points, the criterion to pass as NDE in the strict sense [7]. Under the impression that this instrument may not be sensitive enough to detect experiences associated with a transient shortage of brain oxygen during CA, we included 15 more with detailed recollections from a period near to their CA (Table 1). Together with the strict NDE cases, they consisted of 9 females and 11 males, a sex ratio not significantly different from the other patients (*p* = 0.199). For more detailed interview summaries than in the table, see Supplementary Material File S4.

### 2.6. Statistics

To estimate the probability that cases distributed equally into several categories, we subjected the numbers to a χ^2^ test (chi-squared [15]). By conventional χ^2^ tables (e.g., in the cited book) the limiting probability *p* was obtained. For two categories, R.A. Fisher’s exact test was used, several online calculators all returning precisely the same result.

## 3. Results

### 3.1. Compromised Memories

A few cases suffered severe losses in memories for events preceding their CA (retrograde amnesia). This occurred to several blackout cases, but also four cases with recollections (a similar fraction: *p* = 0.78) had lost at least 1 day from their memory store. Thus, it is possible to keep in mind perceptions during or shortly after a CA while forgetting others preceding the same CA (see [9], p. 180). In contrast, anterograde loss of memory was reported significantly more often by cases with acute recollections. For 10 of these 20 patients, the recovery period took longer than 2 days, but only for 19 of the other 106 patients (*p* = 0.0069, Fisher’s exact test for 2 × 2 contingency tables).

### 3.2. A First Patient Seems to Comment on a Hidden Image

**Figure 2:**
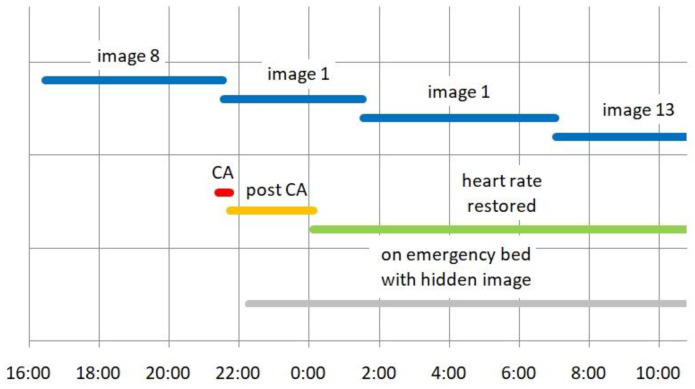
Presentation of randomized pictures visible only from above. Case K (№ 83) suffered CA (red line) in hospital late in the evening. She was rushed to the emergency unit and installed on the bed (gray line) with image presentation to the ceiling (not noticeable from the bed). During the post-CA period (orange line), heart rate in all likelihood still did not support regular brain function. Ordinary rate (green line) was restored more than 2 h after CA. Image presentation (blue lines) had switched during the acute CA from image 8 to image 1 over the empty bed. The next switch occurred 4 h later (with the patient present) from image 1 to image 1 (accidently the same image once more), and another switch 5½ h later from image 1 to image 13. During these latter two periods, brain function had returned to nearly normal and the patient was sleeping. None of the presented images came close to the impression reported later by the patient.

For more than 5 years, a notebook above one emergency bed displayed image after image to the naked ceiling above without any consequences, but in late August 2017, patient K (a 79-y-old female) was installed on the bed (see Figure 2). It took 83 further days until the account of this patient stirred our interest. She had seen a field with beautiful pink flowers resembling water lilies, all of similar size. In her words, this was the first impression “during waking up” and she added: “It was great that the medical staff was capable to display it for me”. When she saw these flowers, she was sure that she would “return”. For the first (and only) time, we had the suspicion that a patient made reference to one of our hidden images. The study design required our patience. Years later, after the closing of the study, we read out the presentation history. The image shown during the acute period (CA and post CA, Figure 2) had not the slightest resemblance to the scenery described by the patient. This may be seen as a negative result, but in fact it vindicated the generally accepted view that consciousness depends solely upon brain function.

### 3.3. Influence of Attitude towards Religious Matters

**Figure 3:**
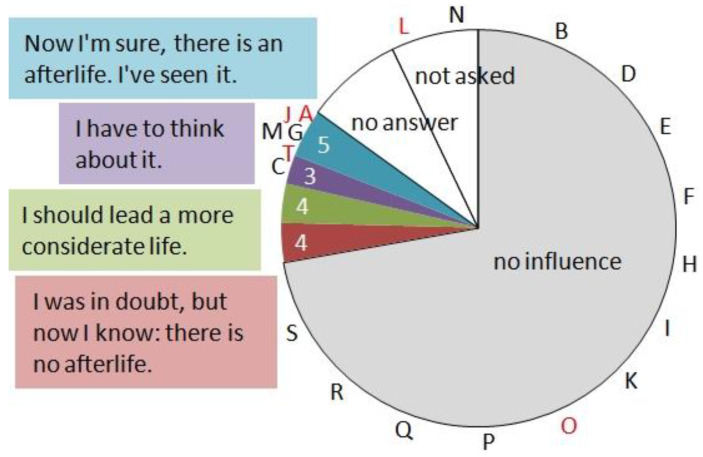
Influence on religious conviction. Patients were asked whether their CA had an influence on their religious conviction. Mostly, believers stayed believers, and non-believers remained in their position. However, 16 cases (the sectors in color) changed their mind. Cases with memories (capitals, in red with ≥7 Greyson points) cluster at one of the sectors.

In the first round of questions, patients were asked about religious conviction and attitude towards central features of belief (questions B5–B10). While the clientele of the main Viennese hospital still adhered in large part to Catholicism (39% of our cohort), the fraction declaring no religion came close to this figure (36%). Four cases with detailed recollections (A, J, O, and T) were of Catholic (A and T), Islamic (O), or no conviction (J). Most subjects (91) ascertained they would have given the same answers before their CA, but a minority of 16 underwent a change in mind (Figure 3). Four cases had revised their position from an indifferent one to a position of clear denial of an afterlife; they were convinced that any post-mortal ‘world’ should have been apparent to them in case it really existed. The last group exclusively consisted of ‘experiencers’, three of them with ≥7 Greyson points. They had doubts before, but were sure now: there is an afterlife. The distribution of capitals over the colored sectors in Figure 3 was heterogeneous (χ^2^ = 13.2, 3 degrees of freedom, *p* < 0.005).

### 3.4. Did You Notice Anything Similar in the Media?

This question in the last section of the questionnaire should safeguard against any bias by media influence (question E3). No such bias was found (χ^2^ 6.46, 6 degrees of freedom, *p* > 0.25). Thirteen ‘experiencers’ were familiar with NDE stereotypes, but four were not, quite close to the situation in the whole sample (65%/22%). An impressive example was case T, a 46-y-old female with a Greyson score of 18 out of 32 possible points, but without ever having heard about NDEs before. Only after her strong experience, she began to search the internet for such reports, with high motivation and interest.

### 3.5. Influence of the Duration of Cardiac Arrest

**Figure 4:**
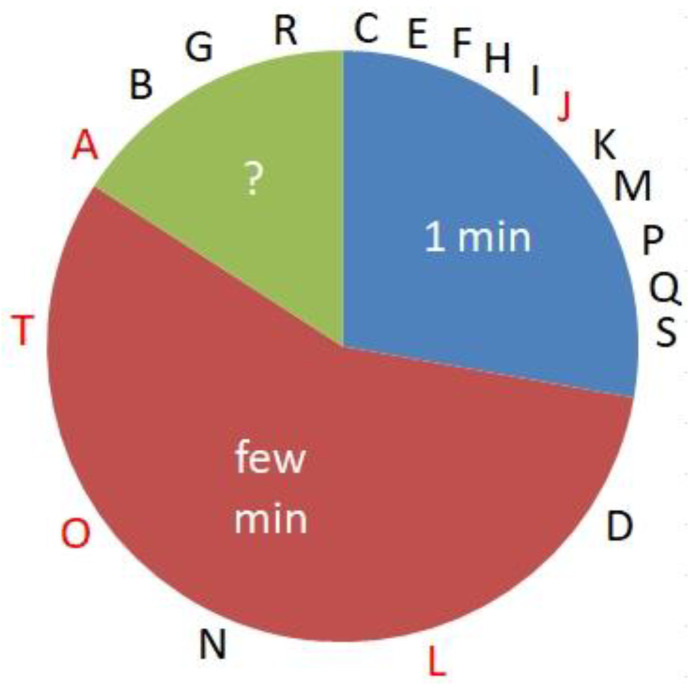
Approximate delay in the onset of CPR measures in 126 CA victims. For 20 cases (16%, ?), no information was available to assign them to sectors ‘1 min’ or ‘few min’. Twenty cases with detailed memories during or shortly after CA (capitals, those with ≥7 Greyson points in red) distributed non-randomly over the three sectors (χ^2^ 10.74, 2 degrees of freedom, *p* < 0.005).

We divided our cases into three groups depending on the circumstances of the event. The first group of episodes occurred in a clinical setting, either in a hospital or during treatment in a professional health institution. Here, we expected a fast routine protocol and the start of CPR measures in about 1 min. The second group was either together with a partner or in a public place, e.g., a tram, tennis court, restaurant, or similar. Here, we assumed that the health condition was quickly recognized, prompting professional input within a few min. Often a close relative was instructed via phone to start resuscitation. Most cases belonged to one of these two groups (see Figure 4). CAs of the third category conformed neither to the first nor the second type. Among those twenty CA victims with detailed recollections, eleven (55%) had received early treatment (first category), while among the rest (n = 106), only 23% belonged to this sort. It was more likely to have detailed recollections if resuscitation had started within 1 min in a healthcare setting than if it was started a few min later in a private or non-professional setting.

## 4. Discussion

In a prospective study based on all patients needing emergency treatment in a large hospital due to CA, we followed up those cases with mental state restored to give a reliable account of their subjective impressions before, after, and possibly also during the incident. During or immediately following CA, only 20 subjects of 126 went through an intriguing experience that remained in their memory. The intensity of this experience was variable and difficult to quantify. A widely used instrument (the Greyson scale) failed to identify some impressive reports as NDE; only five cases passed as NDE in the strict sense. The faster a patient was subjected to CPR measures, the greater the likelihood of a detailed account. In comparison to other studies (Table 2), this study met with a low number of NDE cases in the strict sense. This may reflect the high variability in the frequency of the phenomenon across populations. It may, however, have been of relevance to avoid typical NDE terminology in the communication introducing the project to the patients. This begins with the title of the project (‘Memory processes in CA patients’). About two thirds of the patients were well acquainted with near-death terminology, but apparently this was without influence on their own perception. Nevertheless, some patients reported astonishing episodes that were of high relevance to them, often with symbolic meaning.

Some acute clinical conditions seem to favor subjective experience with qualities indistinguishable from reality. Thonnard et al. [25] subjected 21 patients surviving coma after traumatic brain injury to the Greyson scale and, in addition, to a memory characteristics questionnaire. Eight of them scored at least 7 Greyson points and reported memories with even more characteristics than memories for real events. The authors referred to them as super-real or flashbulb memories, regarding them as hallucinatory or dream-like with a neurophysiological origin and no correlation to events occurring in reality. Additionally, Palmieri et al. [26] provided EEG and questionnaire evidence for the high similarity of NDE memories to memories for real events (and not for imagined events). In comparison to flashbulb memories, NDE memories include more details and episodic/personal information [27] and seem to be more central to the experiencer’s identity. Three of our patients stressed the highly realistic nature of their experience during or shortly after CA (cases A, P, and T); A and T gave a negative answer to question C4 (‘Do you remember a dream-like state?’). Patient P perceived his acute experience as if switching between two ‘different realities’ (he required two CPRs within a few min). Of his two subjective alternatives, we are inclined to accept as true reality only that one accessible also to the emergency staff surrounding him.

As for the paradox of seeing oneself from an apparently extra-corporal point of view (case J), clinical observations at the Geneva University Hospital [28,29] suggest that the subjective feeling of being localized in three-dimensional space is the result of the continuous cooperation between several brain regions that even in the awake state under specific circumstances can fail and lead to paradox subjective impressions. If the transport of oxygen and glucose to the brain is compromised during CA, the common consequence should be the arrest of all activities, leading to immediate loss of consciousness and collapse. In a few cases, during continued resuscitation efforts, a critical amount of blood still rich in oxygen might reach some brain regions, allowing the resumption of activity. If regions involved in the localization of the own body resume activity without appropriate coordination, they transiently may not yield the usual sensory result. Memories of outstanding personal importance such as those concerning deceased close relatives might be among the first to be rekindled by the slow restoration of blood supply during resuscitation efforts, simply because they participate in more networks than less salient ones.

Our observation that detailed impressions were more often reported by patients undergoing resuscitation efforts quickly agrees with the idea that they were triggered by oxygen-loaded blood, reaching targets ready to resume cooperation. Technical improvements may allow in the near future the simultaneous recording of local functional activity in the brain during acute CPR efforts by the application of near-infrared spectroscopy and EEG probes, if they are easy to position and robust against artifacts. Actually, the application of such probes still requires too much attention and may divert from urgent life-saving measures.

None of us can provide definite proof for the existence of any kind of reality. This epistemological fact was brought forward almost 2.4 millennia ago [30] for the first time in written form and has been endorsed by Kant [31] and Popper [32], just to mention some of the more prominent philosophers on that matter. It may be argued that our reality is an ephemeral concept transiently agreed upon on the basis of current knowledge. From time to time, this concept is subject to substantial change [33]. Each of us may adhere to the opinion that the only ‘true reality’ is the subjective feeling we experience at every single instant, although we admit at the same time the privacy of this experience. Keeping in mind these (sometimes conflicting) points of view, we have to decide for ourselves under which circumstances we adopt one or the other.

## Figures and Tables

**Table 1:** Twenty cases with detailed recollections after recovery from CA. Location of CA, age at CA, delay of interview after CA, points scored at the Greyson questions.

№	Location	Age	Delay (d)	Greyson Points	Recollections
24, A	at home	68.1	11	7	Encounter with deceased relatives, daughter signs to her she can go back.
41, B	at home	47.8	34	2	He dreams an actor is in danger, not him; a written message says he would not die.
46, C	tram	67.2	33	0	Dream of upper half of a head watching him from behind a screen; running numbers on a clock.
53, D	at home	52.3	4	2	Reports whiteout (no blackout), feels very fine when waking up a few min later.
58, E	hospital	73.6	10	2	Feeling of a “presence” reassuring nothing bad can happen.
71, F	hospital	69.9	396	1	Dream during waking up: a train takes another track at the moment she opens her eyes.
72, G	near home	55.8	322	5	His body cruising extremely fast in futuristic settings.
74, H	at home	56.3	528	3	Several vivid dreams during prolonged critical period, in one of them features a Thai spirit of death.
75, I	at home	56.3	399	2	Warm wellbeing, bright light in the ambulance.
79, J	hospital	59.2	553	11	Point of view near the bed looking at himself; passes by a garden door while hearing behind the surgeon calling, vivid dreams during waking up.
83, K	hospital	79.2	83	1	Agreeable impression of a meadow with large pink flowers indicating she will come back.
87, L	hiking tour	45.7	272	9 *	Feels drawn into a tunnel shining in all colors (“Rohrpost”), impression of roofs and blue sky.
101, M	hospital	67.7	122	6	First of four dreams interrupted by reanimating physician’s voice, meets deceased relatives (mother, aunt: “Fine you are here!”).
103, N	subway	66.1	81	3 *	Realistic dream of a cemetery with rails branching, heading for life or death.
109, O	at home	47.4	364	8 *	She is in paradise with Allah, bright and warm, she is happy.
111, P	ergometer	54.9	157	3	Silhouettes against bright light, for a moment he opens his eyes to witness his own CPR, returns to the bright light, and is finally reanimated.
115, Q	hospital	52.0	95	2	Realistic impression: she sees herself from behind standing in front of a big wooden door, the door remains closed.
122 R	near home	76.8	63	1	Irrational behavior for 2 weeks, joyful, extremely imaginative, joking, singing.
124, S	at work	39.6	185	0	Haunted by nightmare featuring “bone men” with bones in their hands, threatening her with death.
125, T	at home	46.5	195	18	Finds herself in some kind of antechamber to heaven, bright with blue sky, a stairway leading up; from there, she is sent back by her father (deceased 27 years ago), she still has obligations in her life.

* Greyson points for cases L, N, and O attributed according to their detailed personal account (as, e.g., done by Schwaninger et al. [14]).

**Table 2:** NDE studies in comparison. Number of subjects reporting an NDE in comparison to the total number.

	Total	NDE	
Parnia et al. [16]	63	4	6.3%
Van Lommel et al. [17]	344	23 ^a^	6.7%
Knoblauch et al. [18]	2044 ^b^	82	4.0%
Schwaninger et al. [14]	30	7 ^c^	23%
Greyson [19]	116	11	9.5%
Klemenc-Ketis et al. [20]	52	11	21%
Berger et al. [21]	22	0	<5%
Parnia et al. [10]	101	9	8.9%
Lallier et al. [22]	118	18	15%
Kondziella et al. [23]	1034 ^d^	106	10.3%
Parnia et al. [24]	21	4 ^e^	19%
actual study	126	5	4.0%

^a^ ‘Very deep NDE’ or ‘deep NDE’ (Greyson scale not used). ^b^ Representative survey of the German population. ^c^ Greyson scale applied to detailed patient account. ^d^ Enrolled by crowd-sourcing from 35 populations. ^e^ ‘Explicit memories’.

## Data Availability

More detailed research data are available as files supplementary to this paper.

## Supplementary Materials

The following supporting information can be downloaded at: https://www.mdpi.com/article/10.3390/jcm12051968/s1, File S1: Key data of all 126 CA cases; File S2: Questionnaire in German (printout for interviews); File S3: Questionnaire in English (to be stored as file; contains macros); File S4: Detailed account of 20 cases with recollections after CA.
