# Supplementary material for: Lapses of the Heart: Frequency and Subjective Salience of Impressions Reported by Patients after Cardiac Arrest
Source: J Clin Med. 2023 Mar 2;12(5):1968. doi: 10.3390/jcm12051968 (PMC10004426; doi:10.3390/jcm12051968)
Supplement: Supplementary file 1 [file jcm-12-01968-s001.zip › jcm-2225714-supp-1.pdf]

Key data of all 126 CA cases

№	sex	age at CA	where?	Δ (d)	Greyson score	start of reanimation	circumstances and account leading to entry in column 'start of reanimation'
1	f	61.8	visit	109	1	few min	in company after church, anesthetist (by chance nearby) starts reanimation
2	m	63.5	home	109	1	few min	in company of spouse
3	f	49.3	transp.	8		few min	railway station
4	m	58.3	?	91		?	
5	m	55.2	street	5		?	arrival at emergency unit 26 min after CA
6	m	41.6	street	11		few min	Vienna Marathon
7	m	65.2	home	5		few min	several remitting fade-outs in presence of spouse, the last at emergency unit
8	f	64.3	home	5		?	
9	m	46.3	?	4		?	
10	m	62.5	home	7	1	few min	reanimation started by son
11	m	55.5	car	5		few min	CA during (slow) driving, reanimation started after stopping the car
12	m	42.3	hosp.	4	1	few min	CA at General Hospital (not in clinical area)
13	m	51.9	sport	1		few min	CA during sokker
14	m	48.4	work	21		few min	CA during lunch break with colleagues
15	f	50.1	home	18		few min	CA during sleep, reanimation started by husband
16	f	39.1	?	14		?	
17	m	50.0	hotel	6		few min	reanimation started by spouse and hotel staff
18	m	67.0	ergom.	7		few min	during work-out (no clinical setting)
19	m	54.9	?	38		?	
20	f	51.5	home	38		few min	reanimation started by daughter
21	m	44.1	sport	31		few min	reanimation started by son
22	m	83.3	home	3		1 min	CA at emergency unit
23	m	79.4	home	2		few min	reanimation started by daughter
**A**	f	68.1	home	11	**7**	?	living alone, CA at 11:30 am, arrival at emergency unit 1:30 pm
25	m	52.9	?	27		?	living alone
26	m	58.6	transp.	9		few min	CA in public transport
27	f	67.1	ambul.	67		1 min	CA in ambulance
28	m	66.7	home	44		?	reanimated 3 times
29	m	54.2	street	5		few min	driver, found near his taxi, arrest for 7 min
30	m	71.9	?	16		few min	reanimated after 5 min
31	m	62.8	sport	6		few min	reanimation started on tennis court
32	m	40.7	home	9		few min	reanimation started by spouse
33	f	33.9	work	7		few min	CA in painting class
34	m	70.1	?	12		1 min	unconscious for 2-3 min
35	f	55.2	hosp.	6		few min	CA in non-clinical section of General Hospital
36	m	74.7	hosp.	4		1 min	CA at cardiac station of General Hospital
37	m	55.9	sport	5		1 min	ambulance arrives prior to CA
38	f	84.4	home	4	3	few min	CA in presence of relatives
39	m	54.4	ambul.	6		1 min	CA in ambulance, second CA at emergency unit
40	m	66.1	home	10		1 min	emergency team arrives prior to CA
**B**	m	47.8	home	34	**2**	?	found beside the phone
42	m	53.5	street	10		few min	CA at daytime on busy public place (Kagraner Platz)
43	m	73.0	hosp.	2		few min	CA in General Hospital outside clinical sector
44	m	45.4	ambul.	4	1	1 min	CA in ambulance
45	m	49.3	home	3		1 min	CA after arrival of emergency team
**C**	m	67.2	transp.	33		1 min	CA in streetcar, reanimation by 2 medical doctors accidentally in same carriage
47	f	41.3	home	13		few min	reanimation started by partner
48	m	54.4	ambul.	3	1	1 min	CA in ambulance
49	m	63.1	home	31		few min	reanimation started by spouse
50	m	46.0	home	10		few min	son starts reanimation by instruction by phone
51	f	72.6	home	2		1 min	emergency team arrives prior to CA

52	m	86.1	home	5		?	CA during daytime, 2-person household, ambulance arrives after 15 min
**D**	m	52.3	home	4	**2**	1 min	emergency team arrives during CA
54	m	45.3	work	12		?	security, Friday evening, checks the door of a shop at Westbahnhof, collaps documented by video camera
55	m	62.2	home	5		few min	wife calls emergency team, 7 reanimations prior to, 4 after arrival at hospital
56	m	28.8	ambul.	9		1 min	emergency team arrives prior to CA
57	m	49.8	hosp.	2		1 min	CA at General Hospital prior to inserting stent
**E**	m	73.6	hosp.	10	**3**	1 min	CA during glaukoma OP (Hera hospital)
59	f	65.4	hosp.	7		1 min	CA at cardiac station of General Hospital after drawing blood
60	m	76.3	sport	2		few min	CA during jogging in company
61	m	56.5	hosp.	4	1	1 min	Sunday at noon heart problems; ambulance at 1 pm; at General Hospital at 2 pm; CA at 3 pm, stents inserted
62	m	50.1	sport	11		?	CA during tour on bicycle, found beside his bicycle
63	f	48.9	home	10		few min	first aid by husband and son
64	f	51.0	work	7		few min	CA at work in presence of colleagues
65	m	37.0	work	494		few min	CA at work in presence of colleagues
66	f	75.0	visit	454		few min	CA during eating with friends
67	f	44.5	transp.	439		few min	CA on railway platform
68	m	57.9	transp.	395		few min	CA during driving a streetcar
69	m	69.8	street	413		?	found on sidewalk on a Wednesday evening on his way home from a 'Heuriger'
70	f	76.0	hosp.	298		1 min	first CA at hospital (Rudolfstiftung), further CA at General Hospital
**F**	f	69.9	hosp.	396	**2**	1 min	CA at Emergency Unit of General Hospital after a stay of several days because of pneumonia
**G**	m	55.8	street	322	**5**	?	found outside appartment in staircase
73	m	64.8	visit	272		few min	CA in presence of others
**H**	f	56.3	home	528	**3**	1 min	CA 1 d after admission to General Hospital
**I**	m	66.5	ambul.	399	**2**	1 min	CA in ambulance
76	m	69.2	transp.	189	2	few min	CA in streetcar, first aid personnel accidentally in same carriage, but 6-7 min without support
77	m	55.2	home	267		few min	resuscitation started by spouse
78	m	62.7	street	254		?	walking the dog on early Sunday morning (7 am)
**J**	m	59.2	hosp.	553	**11**	1 min	CA during orthopedic OP (Herz Jesu Hospital)
80	f	71.6	ambul.	97		1 min	CA in ambulance
81	m	62.2	street	72		few min	on way home after church, help within 7-10 min
82	m	66.1	home	62		few min	CA in presence of spouse, ambulance was ordered before
**K**	f	79.2	hosp.	83	**1**	1 min	CA in General Hospital, reanimation starts within 1 min, spontaneous heart activity after 20 min
84	m	58.1	home	144		few min	reanimation started by friends
85	m	51.3	car	80		few min	collapsed during driving, car comes to a stand, resuscitation started by several rescuers, spontaneous heart activity after > 1 h
86	m	57.8	shop	588		1 min	reanimation starts at 0.5 min
**L**	m	45.7	sport	272	**9**	few min	in company at the beginning of a hiking tour
88	m	57.9	ergom.	74	1	1 min	CA on ergometer at cardiologist
89	f	50.8	home	327		few min	first aid by partner
90	f	37.1	work	91		few min	CA in presence of several others, resuscitation started following instruction by phone
91	m	70.3	home	75	1	few min	CA in presence of colleague
92	m	62.9	home	477		few min	CA during sleep, reanimation started by spouse
93	m	56.8	sport	28		few min	run at Silvester in company of several others
94	m	39.9	home	59		few min	resuscitation started by spouse
95	m	75.8	hosp.	121		few min	CA prior to entering the General Hospital

96	m	62.1	home	130		few min	resuscitation started by daughter following instruction by phone
97	m	19.9	home	121		few min	first aid by neighbor
98	m	50.0	hosp.	92		1 min	CA in General Hospital during insertion of catheter, several repetitive reanimations
99	m	51.4	sport	115		few min	reanimation after accident in public swimming pool
100	m	70.7	visit	107		few min	CA at festivity with family (including dancing)
**M**	m	67.7	hosp.	122	**6**	1 min	CA at General Hospital during scan of lymph nodes
102	m	71.6	home	60		few min	resuscitation started by spouse according to instruction by phone
**N**	f	66.1	transp.	81	**3**	few min	CA in underground, resuscitation started by young medical doctor in same carriage
104	m	65.0	home	344		few min	first aid by uncle
105	f	75.4	street	153		few min	CA at 8 am in front of house (3rd district, busy area), heart stopped 4 times in 20 min
106	f	48.2	street	117	1	few min	CA during a walk in company in the evening at the Donaukanal
107	m	55.8	sport	172	1	1 min	CA at arrival of emergency team
108	f	28.0	home	94		few min	CA during sleep, resuscitation started by husband, fast arrival of emergency team
**O**	f	47.4	home	364	**8**	few min	CA during sleep, resuscitation started by husband, fast arrival of emergency team
110	m	50.9	street	114		?	found on street near his appartment in early evening
**P**	m	54.9	ergom.	157	**3**	1 min	falls off the ergometer into the arms of medical doctor
112	f	28.8	home	116		few min	CA at Sunday morning in presence of fiancé
113	m	50.6	home	311		few min	resuscitation started by spouse according to instruction by phone
114	m	59.2	home	64		?	falls off from bed during CA into narrow gap, girlfriend is unable to recover him → delay of resuscitation measures
**Q**	f	52.0	hosp.	95	**2**	1 min	comes to General Hospital with shoulder pain, CA during infiltration
116	m	38.1	street	250		few min	CA in the middle of a crossroad
117	m	66.5	visit	87		few min	CA at 'Heuriger' in company
118	m	53.2	home	113		few min	reanimation started by girlfriend
119	m	91.0	home	185		1 min	emergency team arrives prior to CA
120	m	30.4	home	148		?	called his cousin by phone because he felt ill
121	m	58.7	sport	365		few min	CA at dancing school in company
**R**	m	76.8	street	63	**1**	?	walking the dog in quiet outskirt area
123	m	69.2	home	71		few min	resuscitation started by spouse; rescue team 7 min, spontaneous heart activity 17 min after CA
**S**	f	39.6	ambul.	185		1 min	first CA in ambulance, next at General Hospital
**T**	f	46.5	home	195	**18**	few min	partner starts resuscitation following instruction by phone
126	f	64.4	hosp.	137		1 min	CA at General Hospital during dialysis

bold: remarkable recollections (**red**: Greyson ≥ 7), majuscule instead of №

green background: short version of interview

Δ (d): time lag between CA and interview (days)

ambul., ambulance; ergom., ergometer; hosp., hospital; transp., public transport; ?, not known
