# Supplementary material for: Lapses of the Heart: Frequency and Subjective Salience of Impressions Reported by Patients after Cardiac Arrest
Source: J Clin Med. 2023 Mar 2;12(5):1968. doi: 10.3390/jcm12051968 (PMC10004426; doi:10.3390/jcm12051968)
Supplement: Supplementary file 1 [file jcm-12-01968-s001.zip › jcm-2225714-supp-2.pdf]

GEDÄCHTNISLEISTUNGEN VON HERZSTILLSTAND-ÜBERLEBENDEN

INTERVIEW–FORMULAR, Druckvorlage

Nach Wiederherstellung der Herzfunktion auf der Notfall- bzw. Intensivstation, wird der Patient zur weiteren Behandlung auf die Routinestation transferiert. Das Interview wird dort nach Rücksprache mit dem behandelnden Arzt geführt oder zu einem späteren Zeitpunkt (nach der Entlassung). Nur solche Patienten werden kontaktiert, deren Zustand es ihnen erlaubt selbst zu entscheiden, ob sie an der Befragung teilnehmen wollen oder nicht.

Der Interviewer übergibt dem Patienten die *Zustimmungs-Erklärung*. Entweder liest er sie mit dem Patienten gleich durch, oder er hinterlässt sie und kommt zu einem späteren Zeitpunkt wieder. Womöglich möchte der Patient den Inhalt mit dem Arzt oder einem Angehörigen besprechen. Angehörige dürfen beim Interview dabei sein.

Der Fragebogen hat folgende Abschnitte:

**A**	Formale Daten	Name, Geburtsdatum, Datum des Stillstands und des Interviews
**B**	Daten zur Person	Ausbildung, Lebensumstände, Religion
**C**	Akute Erinnerungen	Eindrücke vor, während, und nach dem Herzstillstand
**D**	Greyson	Greyson NDE scale
**E**	Abschließende Fragen	Vorwissen, Gedächtnisprobleme

Wirkt der Patient erschöpft, soll die Befragung unterbrochen werden. Klagt der Patient über Gedächtnisprobleme, schlägt man ihm Tests diverser Gedächtnisleistungen vor (siehe Frage D4). Das vollständige Interview wird später in ein elektronisches Format übertragen (in Englischer Sprache) um die Kommunikation mit anderen teilnehmenden Arbeitsgruppen zu ermöglichen (die Papierversion bleibt im Haus).

A: Formale Daten

Name:	
Geburtsdatum:	
Datum des Stillstands:	
Ort des Stillstands:	
Datum des Interviews:	
Ort des Interviews:	

B: Persönliche Daten

1) Wo leben Sie?

- ☐ Auf dem Land
- ☐ Kleinstadt (< 100.000)
- ☐ Großstadt (> 100.000)

2) Welche Ausbildung hatten Sie?:

- ☐ Pflichtschule
- ☐ Lehre
- ☐ Matura
- ☐ Universität
- ☐ anderes: ______________________________

3) Was ist ihre Beschäftigung?

Bitte beschreiben Sie kurz: ______________________________

Bei Ruhestand frühere Beschäftigung: ______________________________

4) Wohnverhältnisse

- ☐ Ich wohne allein.
- ☐ Ich wohne in einem 2-Personen-Haushalt.
- ☐ Mein Haushalt besteht aus 3 oder mehr Personen.
- ☐ Institution (Heim, betreutes Wohnen, ...)

5) Betrachten Sie sich als einer bestimmten Religion zugehörig?

- ☐ Yes
- ☐ No

5a) Wenn "Ja": welcher? ______________________________

6) Abgesehen von sozialen Ereignissen wie Hochzeiten, Begräbnissen oder Taufen, wie oft nehmen Sie z.Z. an religiösen Zeremonien teil?

- ☐ Mindestens einmal pro Woche
- ☐ Ein paar Mal im Jahr
- ☐ Einmal im Jahr
- ☐ Nie oder fast nie

7) unabhängig davon, ob Sie einen religiösen Ort wie Kirche, Moschee, Synagoge oder Tempel besuchen oder nicht: würden Sie sich wie folgt bezeichnen?

- ☐ spirituell
- ☐ religiös
- ☐ agnostisch
- ☐ überzeugter Atheist
- ☐ anders: ______________________________

8) Woran glauben Sie?

- ☐ Gott
- ☐ Religiöse Figur: ______________________________
- ☐ Leben nach dem Tod
- ☐ Seele
- ☐ Etwas anderes, nämlich: ______________________________

9) Wenn Sie sich ein Weiterleben über den Tod hinaus vorstellen: Was erwarten Sie?

- ☐ Ich werde aufgefordert, für meine Handlungen Rechenschaft abzulegen.
- ☐ Ich erwarte, dass mein Körper aufersteht.
- ☐ Ich erwarte, meine verstorbenen Verwandten zu treffen.
- ☐ Anderes, nämlich: ______________________________

10) Hat sich Ihre Erwartung durch den Herzstillstand geändert

- ☐ Nein
- ☐ Ja, nämlich: ______________________________

C: Freie Assoziationen rund um den Herzstillstand

1) Was ist das Letzte, woran Sie sich aus der Zeit VOR dem Herzstillstand erinnern?

2) Hatten Sie irgendwelche Eindrücke WÄHREND der akuten Phase?

3) Was ist das Erste, das Sie NACH Ihrem Herzstillstand bewusst wahrgenommen haben?

4) Erinnern Sie sich an einen traumähnlichen Zustand?

D: Greyson Scale (0, 1, or 2 beziehen sich auf die Intensität der Eindrücke).

Die folgenden Fragen beziehen sich auf mögliche Eindrücke oder Gefühle während eines Herzstillstands. Auch wenn im Abschnitt C nichts berichtet wird, könnte die konkrete Erwähnung bereits Vergessenes wieder ins Gedächtnis rufen. (Kein Eintrag gilt auch als ‚Nein'.)

1) Hatten Sie das Gefühl, dass alles schneller oder langsamer als sonst ablief?

- ☐ 0 = Nein
- ☐ 1 = Die Eindrücke schienen schneller oder langsamer als normal abzulaufen
- ☐ 2 = Alles schien gleichzeitig zu geschehen; oder die Zeit verlor jede Bedeutung.

2) Haben sich Ihre Gedanken beschleunigt?

- ☐ 0 = Nein
- ☐ 1 = Sie haben sich merklich beschleunigt.
- ☐ 2 = Sie haben sich unglaublich stark beschleunigt.

3) Hatten Sie das Gefühl, frühere Erlebnisse noch einmal zu erleben?

- ☐ 0 = Nein
- ☐ 1 = Ich wurde an einige frühere Ereignisse erinnert.
- ☐ 2 = Meine Vergangenheit lief vor mir ab, ohne dass ich es kontrollieren konnte.

4) Hatten Sie plötzlich das Gefühl, alles zu verstehen?

- ☐ 0 = Nein
- ☐ 1 = Alles über mich oder andere
- ☐ 2 = Alles über die ganze Welt

5) Hatten Sie ein Gefühl von Frieden und Wohlwollen?

- ☐ 0 = Nein
- ☐ 1 = Erleichterung und Ruhe
- ☐ 2 = Unglaublicher Friede und Wohlwollen

6) Hatten Sie ein Gefühl von Freude?

- ☐ 0 = Nein
- ☐ 1 = Ich fühlte mich glücklich.
- ☐ 2 = Ich empfand unglaubliche Freude.

7) Hatten Sie ein Gefühl von Harmonie und Einheit mit der Welt?

- ☐ 0 = Nein
- ☐ 1 = Ich fühlte keinen Konflikt mehr mit der Natur.
- ☐ 2 = Ich fühlte mich verbunden oder vereint mit der Welt.

8) Sahen Sie strahlendes Licht, oder fühlten Sie sich davon umgeben?

☐ 0 = Nein
☐ 1 = Ein ungewöhnlich helles Licht
☐ 2 = Alles war von einem übernatürlichen Licht erfüllt.

9) Waren Ihre sensorischen Empfindungen lebhafter als normal?

☐ 0 = Nein
☐ 1 = Lebhafter als normal
☐ 2 = Viel intensive als normal

10) Haben Sie Dinge wahrgenommen, die Sie normalerweise gar nicht wahrnehmen hätten können?

☐ 0 = Nein
☐ 1 = Ja; ich weiß aber nicht, ob die Wahrnehmung richtig war.
☐ 2 = Ja; und es hat sich bestätigt, dass die Wahrnehmung richtig war.

11) Hatten Sie den Eindruck, in die Zukunft zu sehen?

☐ 0 = Nein
☐ 1 = Ja, in meine persönliche Zukunft
☐ 2 = Ja, in die Zukunft der Welt

12) Hatten Sie das Gefühl, sich von Ihrem Körper zu trennen?

☐ 0 = Nein
☐ 1 = Ich habe meinen Körper nicht mehr wahrgenommen.
☐ 2 = Ich habe meinen Körper verlassen und befand mich außerhalb von ihm.

13) Hatten Sie das Gefühl, in eine andere, nicht-irdische Welt zu gelangen?

☐ 0 = Nein
☐ 1 = Ich kam an einen unbekannten, fremden Ort.
☐ 2 = Ich kam an einen übernatürlichen, geheimnisvollen Ort.

14) Hatten Sie das Gefühl, ein mystisches Wesen zu treffen, oder eine unbekannte Stimme zu hören?

☐ 0 = Nein
☐ 1 = I hörte eine Stimme, die ich nicht zuordnen konnte.
☐ 2 = Ich traf ein bestimmtes Wesen, oder hörte eine Stimme nicht-irdischer Herkunft.

15) Haben Sie Verstorbene gesehen, oder religiöse Geister?

☐ 0 = Nein
☐ 1 = Ich fühlte ihre Gegenwart.
☐ 2 = Ich habe sie wirklich gesehen.

16) Sind Sie an eine Grenze gelangt, an einen Punkt ohne Wiederkehr?

☐ 0 = Nein
☐ 1 = Ich habe mich bewusst zu einer Rückkehr ins Leben entschieden.
☐ 2 = Ich kam an eine Grenze, die ich nicht überqueren durfte, oder wurde gegen meinen Willen zurückgeschickt.

Greyson-Gesamtwert: ☐ **/32**

E: Abschließende Fragen

1) Hat Sie Ihr Herzstillstand an ein früheres Erlebnis erinnert?

☐ Ja ☐ Nein ☐ Unklar

1a) Falls „Ja“: Beschreiben Sie bitte genauer.

2) Hat schon einmal jemand aus Ihrer Familie oder Ihrem Bekanntenkreis von ähnlichen Eindrücken berichtet?

☐ Ja ☐ Nein ☐ Unklar

2a) Falls „Ja“: Beschreiben Sie bitte genauer.

3) Haben Sie schon aus den Medien von solchen Eindrücken gehört?

☐ Ja ☐ Nein ☐ Unklar

3a) Falls „Ja": Beschreiben Sie bitte genauer.

4) Haben Sie den Eindruck, dss sich Ihre Wahrnehmung oder Ihre Merkfähigkeit seit dem Herzstillstand verändert hat?

☐ Ja ☐ Nein ☐ Unklar

4a) Falls „Ja“: Beschreiben Sie bitte genauer.

4b) Falls „Ja“: Ein Gedächtnistest knnte sinnvoll sein. Wären Sie damit einverstanden?

☐ Ja ☐ Nein ☐ Will noch überlegen

Das interview führte:

Ort: **Datum:**
