# Supplementary material for: Lapses of the Heart: Frequency and Subjective Salience of Impressions Reported by Patients after Cardiac Arrest
Source: J Clin Med. 2023 Mar 2;12(5):1968. doi: 10.3390/jcm12051968 (PMC10004426; doi:10.3390/jcm12051968)
Supplement: Supplementary file 1 [file jcm-12-01968-s001.zip › jcm-2225714-supp-3.docm]

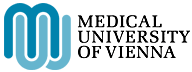
**MEMORY PROCESSES IN CARDIAC ARREST PATIENTS**

INTERVIEW SCHEDULE, electronic form

As soon as cardiac function has been stabilized at the emergency and the intensive care unit, the patient is transferred to a routine station of the hospital for treatment of the underlying condition (internal medicine). Before contacting the patient, the treating physician should be approached to learn about the actual condition of the patient. If this condition allows, the patient is asked for his/her agreement to participate in the study. Only patients in a condition allowing them eventually to decline participation should be contacted.

The interviewer should hand over to the patient the *patient agreement form*. He can read and explain the content to the patient immediately, or he leaves the patient with the form to come back the next day, if the patient prefers to read carefully; the patient may also wish to discuss the project with a relative. Relatives may be present during the interview.

The questionnaire has the following sections:

| **A** | Formal data | Name, date of birth, date of cardiac arrest, date of interview |
| --- | --- | --- |
| **B** | Personal data | Education, living condition, religion |
| **C** | Memories around CA | Last memory before, impressions during, first memory after CA |
| **D** | Greyson | Greyson NDE scale |
| **E** | Concluding questions | Pre-knowledge, cognitive problems |

If the patient is tired and needs to rest, schedule a follow-up interview. If the patient leaves the hospital prior to contact or to completing the interview, schedule a meeting to complete the interview. If the patient suffers from cognitive problems (see question D4), standard follow-up tests should be arranged (in agreement with the patient) concerning various memory faculties. After completing the interview, results are transferred into electronic form (in English language) for easy compilation of data from all participating sites (printed forms stay at the local site).

**A: Formal Data**

| Name: |  |
| --- | --- |
| Date of birth: |  |
| Date of cardiac arrest: |  |
| Location of cardiac arrest: |  |
| Date of interview: |  |
| Location of interview: |  |

**B: Personal Data**

**2) Where do you live**?

**2) Education**:

|  |  |
| --- | --- |

**3) What is your occupation?**

| Please state: |  |
| --- | --- |
| If retired, what was your occupation? |  |

**4) Living conditions:**

**5) Do you regard yourself as belonging to any particular religion?**


| **5a) If yes**: to which one? |  |
| --- | --- |

**6) Apart from weddings or funerals, how often do you attend religious services?**

**7) Independently of whether you attend religious services or not, would you rate yourself…**

|  |  |
| --- | --- |

**8) Which if any of the following do you believe in?**

|  |  |
| --- | --- |

|  |  |
| --- | --- |

**9) If you believe in an after-life, which of the following would describe your beliefs?**

|  |  |
| --- | --- |

**10) Has your expectation changed after the cardiac arrest?**

|  Yes; that is: |  |
| --- | --- |

**C: Memories around CA**

**1) What is your last memory BEFORE your cardiac arrest?**

**2) Do you remember anything from the time DURING your unconsciousness?**

**3) What was your first impression AFTER your cardiac arrest?**

**4) Do you remember a dream-like state?**

**D: Greyson Scale** (0, 1, or 2 refer to the intensity of the impression).

The following questions refer to eventual perceptions or feelings during CA. Even if the patient did not indicate any such impressions (C2), mentioning may spontaneously remind the patient of sensations he/she had forgotten. Form starts with zero in all fields to the left (avoid empty fields).

|  | **1) Did you have the impression that everything happened faster or slower than usual? (did time seem to speed up or slow down?)** | |
| --- | --- | --- |
| 0 | 0 = No | |
|  | 1 = Everything seemed to go faster or slower than usual | |
|  | 2 = Everything seemed to be happening at once; or time stopped or lost all meaning | |
|  |  | |
|  | **2) Were your thoughts speeded up?** | |
| 0 | 0 = No | |
|  | 1 = Faster than usual | |
|  | 2 = Incredibly fast | |
|  |  | |
|  | **3) Did scenes from your past come back to you?** | |
| 0 | 0 = No | |
|  | 1 = I remembered many past events | |
|  | 2 = My past flashed before me, out of my control | |
|  |  | |
|  | **4) Did you suddenly seem to understand everything?** | |
| 0 | 0 = No | |
|  | 1 = Everything about myself or others | |
|  | 2 = Everything about the whole world | |
|  |  | |
|  | **5) Did you have a feeling of peace or pleasantness?** | |
| 0 | 0 = No | |
|  | 1 = Relief or calmness | |
|  | 2 = Incredible peace or pleasantness | |
|  |  | |
|  | **6) Did you have a feeling of joy?** | |
| 0 | 0 = No | |
|  | 1 = Happiness | |
|  | 2 = Incredible joy | |
|  |  | |
|  | **7) Did you feel a sense of harmony or unity with the universe?** | |
| 0 | 0 = No | |
|  | 1 = I felt no longer in conflict with nature | |
|  | 2 = I felt united or one with the world | |
|  |  | |
|  | **8) Did you see, or feel surrounded by, a brilliant light?** | |
| 0 | 0 = No | |
|  | 1 = An unusually bright light | |
|  | 2 = A special light conveying a super-natural feeling | |
|  |  | |
|  | **9) Were your senses more vivid than usual?** | |
| 0 | 0 = No | |
|  | 1 = More vivid than usual | |
|  | 2 = Incredibly more vivid | |
|  |  | |
|  | **10) Did you seem to be aware of things going on that normally should have been out of sight from your actual point of view as if by extrasensory perception?** | |
| 0 | 0 = No | |
|  | 1 = Yes, but it was not checked out if these impressions were correct | |
|  | 2 = Yes, and it later turned out that these impressions were correct | |
|  |  | |
|  | **11) Did scenes from the future come to you?** | |
| 0 | 0 = No | |
|  | 1 = Scenes from my personal future | |
|  | 2 = Scenes from the world’s future | |
|  |  | |
|  | **12) Did you feel separated from your body?** | |
| 0 | 0 = No | |
|  | 1 = I lost awareness of my body | |
|  | 2 = I clearly left my body and existed outside it | |
|  |  | |
|  | **13) Did you seem to enter some other, unearthly world?** | |
| 0 | 0 = No | |
|  | 1 = Some unfamiliar and strange place | |
|  | 2 = A clearly mystical or unearthly realm | |
|  |  | |
|  | **14) Did you encounter a mystical being or presence, or hear an unidentifiable voice?** | |
| 0 | 0 = No | |
|  | 1 = I heard a voice I could not identify | |
|  | 2 = I encountered a definite being, or a voice clearly of mystical or unearthly origin | |
|  |  | |
|  | **15) Did you see deceased or religious spirits?** | |
| 0 | 0 = No | |
|  | 1 = I sensed their presence | |
|  | 2 = I actually saw them | |
|  |  | |
|  | **16) Did you come to a border or point of no return?** | |
| 0 | 0 = No | |
|  | 1 = I came to a definite conscious decision to return to life | |
|  | 2 = I came to a barrier I was not permitted to cross; or was sent back against my will. | |
|  |  | |
| 0 | **/ 32 = Total Greyson Score** | Select and press F9 for actualization |

**E: Concluding Questions**

**1) Did your cardiac arrest remind you of some experience in your past**?

1a) If “Yes”: please describe.

**2)** Did any of your **relatives or friends** describe such experiences in connection to a life-threatening incidence?

2a) If “Yes”: please describe.

**3) Did you hear from the media of similar impressions?**

**3a) If “Yes”**: please describe.

**4)** Have your **sensory** or **memory abilities** changed since your cardiac arrest?

**4a) If “Yes”**: please describe.

**4b)** **If “Yes”**: are you interested in performing a **cognitive test**?

**Interviewer:**

**Site: Date:**
