# Supplementary material for: Lapses of the Heart: Frequency and Subjective Salience of Impressions Reported by Patients after Cardiac Arrest
Source: J Clin Med. 2023 Mar 2;12(5):1968. doi: 10.3390/jcm12051968 (PMC10004426; doi:10.3390/jcm12051968)
Supplement: Supplementary file 1 [file jcm-12-01968-s001.zip › jcm-2225714-supp-4.pdf]

Detailed account of 20 cases with recollections after CA

Case **A** (№ 24), a female 68 years old when CA occurred, lives alone after the loss of her husband and one of her two daughters. She collapsed shortly before noon at or near her apartment (witnessed by a neighbor) and arrived at the emergency unit about 2 h later. Transient restoration of cardiac function had already been achieved at the site of her collapse, but further measures were necessary at the hospital. Eleven days after the episode (still in hospital) she underwent our structured interview. Already during the introductory part (What is your religion? Do you believe in God? In a life after death?) she stated, with rising emotion that now, after her experience, she believed in a life after death. She described a bright light and that she has seen her deceased husband and daughter. Both were very beautiful. Her daughter signaled to her (without words) that she was allowed to return to life. During this encounter she was full of joy. The experience generated a more realistic feeling than a common dream. She regained consciousness about 4 h after her collapse. She reached **7** Greyson points: 2 (6) + 1 (8) + 1 (13) + 2 (15) + 1 (16) (question № in parentheses - see supplementary material 2, section D).

Case **B** (№ 41), a male 47 years old when CA occurred, lives alone. On the day of his CA he experienced difficulties in breathing and called the ambulance. He was found near the phone. The interview was conducted 34 d later still in hospital. **B** stated to have been without consciousness for 10-14 d. He underwent experiences during that time, but their chronological sequence remains unclear to him. More generally it seems he was aware to be in a coma, still alive, without worrying about it. More specifically he remembers a dream being someone else (an actor). The reason for his optimism was a written message saying that he was not to die. Greyson points: 1 (2) + 1 (9) = **2**.

Case **C** (№ 46), a male 67 years old when CA occurred, was reanimated in a tram when returning from an exhibition in the Belvedere. He remembers a cigarette in the park, but not how he got into the tram. On the occasion of his interview 33 d later still in hospital he reports a strange encounter with a person unknown to him. The scene was more realistic then a common dream. Visible to him was only the upper part of the head, with eyes that were watching him, with curly hair, and with an oval silver plate on top. The plate was connected to the ceiling by a tube. **C** guesses that some elements of this impression may have been prompted by instruments at the emergency unit. Next to this face he saw a clock with running numbers, accompanied by the sound of bubbling water. The mysterious observer seems to intrigue the patient still after several weeks. **C** is an agnostic. His Greyson score was zero.

Case **D** (№ 53), a male 52 years old when CA occurred is, as a journalist, well acquainted with the topic *cardiac arrest*. On a Saturday around noon, after having visited the Vienna flea market, he feels a strong pain in his chest and knows what that means. His wife calls the ambulance. Immediately after arrival the patient fainted. Four days later, during the interview at the hospital, he described this moment not as a blackout, but as a ‘white-out’. He was told that his heart stopped beating for only about 4 min. When he regained consciousness lying on the carpet in his living room, he enjoyed this moment as very agreeable and felt like after a long refreshing sleep. Greyson points: 1 (5) + 1 (6) = **2**.

Case **E** (№ 58), a male 73 years old when CA occurred, underwent glaucoma surgery at clinic Hera when his heart stopped. The last word he remembers was “intubate”. He was transferred to the emergency unit of the general hospital (nearby). Asked 10 d later about any feelings during his CA, he reported the notice of a “presence”, as if a protecting hand was on his shoulder. He was reminded of an incident decades ago during repair work at a power station when he was at risk losing a leg; he saw a beam of light and felt no pain any more. When regaining consciousness at the emergency unit

of the general hospital, he noticed turbulent activity around him when keeping his eyes closed that immediately stopped when he opened them again. Greyson points: 1 (3) + 1 (5) + 1 (15) = **3**.

Case **F** (№ 71), a female 69 years old when CA occurred, had her interview in the middle of January. Around Christmas she had suffered a severe health crisis. It was not last Christmas but the before-last, and the interview took place more than a year after the CA. She had been transferred to the General Hospital with severe pneumonia. After 10 days she reached a mentally clouded state with high fever and tachycardia. She suffered her CA in this critical condition at the emergency unit. During the period of recovery she had a dream. She was one of the passengers in a train with open carriages. Rails led in a spiral down a conical hill and the train dashed at very high speed down the track. At the foot of the hill, the course split in two directions. **F** thought, the train would take the left turn, but in fact it took the right one and – “Zack!” – she woke up. Emergency staff gathered around her bed and asked her: “How are you?” Greyson point: **1** (1).

Also case **G** (№ 72), a male 55 years old when the CA occurred, suffered his initial CA a long time ago (nearly one year). After coming home from work he experienced difficulties to breathe. He tried to contact his neighbor, but she was not at home. He was found in the staircase lying on the floor. He suffered at least 2 further CAs in the hospital during the following weeks. His cognitive state was unstable during this time, with clouded impressions of his own body moving very fast in a futuristic setting (**G** is reminded of certain computer games, without naming any in particular). Among the questions touching topics of religion is also this one: Do you believe in life after death? **G** did not, but now he does. Greyson points: 2 (1) + 1 (9) + 1 (12) + 1 (13) = **5**.

Case **H** (№ 74), a female 56 years old when the CA occurred, is native from Thailand. Neither in German nor in English direct fluent communication was possible. The interview was accomplished with the help of her (Austrian) husband. The CA happened nearly 1½ years ago as consequence of a health crisis involving not only the heart, but also pancreas and kidneys. **H** spent more than 3 months in the general hospital. Her crisis was accompanied by many dreams. Her husband and I managed to understand at least some of them. The most impressive one was the following: There seems to exist in old Thai mythology a character responsible for leading the dying away from life to death. **H** saw this God standing beside her bed, apparently waiting for her. To her relief, it was false alarm and the sinister apparition disappeared empty-handed. Greyson points: 1 (9) + 2 (14) = **3**.

Also the CA of case **I** (№ 75), a male 56 years old when the CA occurred, preceded the date of the interview by more than one year. CA is heralded by strong chest pain. The emergency team records an ECG and suggests immediate transfer to a hospital. Mounting the ambulance, he is urged to swallow a few pills. Thereafter – the ambulance still standing in front of the house – the patient switches to “a state of warm well-being”; the “prevailing color was a shining white” (in the patient’s words). Next he hears the voice of the emergency doctor: “Do you hear me?” - And he replies: “Ah… now I was inattentive for a moment…” – “I think so; we just reanimated you.” The patient reported no further incidents at the hospital. Greyson points: 1 (5) + 1 (8) = **2**.

Case **J** (№ 79), a male 59 years old when the CA occurred, was interviewed 1½ years after his CA. His heart stopped during surgery at an orthopedic hospital in Vienna (Herz-Jesu Hospital); the incident was managed in-house (not at the General Hospital). **J** claims that he was standing upright about one meter beside the OP table on which he was supposed to lie, and to observe from there the scenery. However, when asked during the interview how the person lying on the table did look like, he could not provide any information. While apparently standing there, he heard the surgeon 3-4 times calling his name and ordering him to wake up (surgeon and personnel later agreed with this notion). But **J** at this time was not interested in waking up. He felt urged by some kind of ‘energy’ to walk away. He saw a beautiful landscape with flowers in a meadow. He passed a garden door, and the operation

theatre behind him disappeared. In far distance, he noticed a mighty mountain: the Kilimanjaro? He would have liked to walk into this direction, but again the mysterious energy interfered, this time directing his steps back to the OP, once more crossing the garden door, this time in the other direction. **J** spent several further weeks in hospital (he attracted pneumonia). During this critical period he went through several dreams. One of them included a train, with deep night to the right and bright day to the left side. In another dream, little animals (he mentioned a penguin) bring energy down to selected humans on earth. The energy comes from clouds with flashes of lightning under the ceiling of a high room. In the introductory part of the questionnaire, **J** stated not to believe in God. Since the described experiences he at least believes into a guiding energy. Greyson points: 1 (2) + 2 (5) + 1 (6) + 2 (10) + 2 (12) + 1 (13) + 2 (16) = **11**.

Due to difficulties in breathing, case **K** (№ 83), a female 79 years old when the CA occurred, was originally entered as pulmonology patient at the general hospital. The CA happened during her first night there. She was successfully resuscitated and transferred to the emergency unit for further treatment. During the interview 83 d later at home, she surprised her husband (who participated) with the revelation that after losing consciousness she had the agreeable impression of a beautiful meadow with wonderful flowers. The flowers were pink and reminded her of water lilies. Was it a dream? No, she prefers the term 'impression'; she was "pleased that the clinical staff was able to produce it for her". She likes this memory: "Now I knew: I will come back." (See Fig. 2) Greyson point: **1** (6).

For case **L** (№ 87), the short interview version was applied as a telephone call. **L** was later told that he collapsed before the beginning of a hiking tour (after leaving the taxi). He himself has no recollections of this morning and not of the greatest part of the preceding day. Surprisingly, this patient has recollections of the unconscious condition. It began with being "full of love", floating in complete darkness. This 1st state was replaced by uncertainty and rising concern, that something very different was going to happen. Finally, a 3rd state broke through in a very dynamic way. **L** felt drawn into a tunnel shining in all colors. The patient used the German word "Rohrpost" (pneumatic post). For a short time he saw the blue sky and roofs from above. He slipped with high speed through these impressions and finally opened his eyes. He saw the ceiling above his emergency bed at the General Hospital. Without asking the questions in particular, we inferred the following Greyson points from his account: 1 (1) + 2 (2) + 1 (5) + 1 (6) + 1 (10) + 2 (12) + 1 (16) = **9**.

Right from the beginning of this interview, it became clear that case **M** (№ 101), a male 67 years old when the CA occurred, had more to tell than most others. Although **M** was not religious, never attended any ceremonies, and described himself as an agnostic, he now believes in God and in an afterlife. What changed his mind? Fortunate for us (and for him), he survived his CA at the general hospital. In addition (the secret dream of every true scientist): he did so in a CT scanner. Slight drop of bitterness: the target was not the brain, but his lymph nodes. Although 122 d had passed since the CA, **M** still remembered several dreams. Apparently, they left a strong impression. Most relevant for our topic was the first one. He saw his mother and his aunt (both deceased some time ago) in white outfit, laughing happily ("Fine that you're here"). The serene atmosphere was intruded by a female voice: "No, no, he comes later", similar to the voice of the reanimating doctor. Greyson points: 1 (5) + 1 (6) + 1 (13) + 2 (15) + 1 (16) = **6**.

Case **N** (№ 103), a female 67 years old when her CA occurred, was contacted by phone applying the short version of the interview. Eighty one days before, she had collapsed in a Viennese subway. Fortunately, a young physician was in the same carriage. The patient had a realistic dream, with colors, emotions and scents. One feature accompanying the dream was the scent of dry needles fallen from pine trees, as typical for southern cemeteries. **N** was looking for the grave of her mother (of Croatian origin). A railway crossed the area, forging in two directions. For **N** it felt like the decision

between life and death. She had a feeling of omniscience. We attributed **3** Greyson points, two for the omniscience (4) and one for the symbolic representation of a border between life and death (16).

It took exactly one year to arrange an interview with case **O** (№ 109), a muslima 47 years old at the time of her CA. It was worth the patience. During or after her CA, **O** was (according to her account) in paradise with Allah. Everything was fine, she was happy. While this information sounds interesting to us, it feels rather embarrassing to muslims. The interview took place in the general hospital, in the waiting area of the cardiology department. No other patients were around, but nevertheless the couple evoked the impression to participate in something dishonorable. The conversation was kept as short as possible (omitting the laborious Greyson questions). A total of **8** Greyson points were attributed according to her account: 1 (5) + 1 (6) + 1 (8) + 2 (13) + 2 (14) + 1 (16). Surprisingly, at the end **O** revealed that 25 years ago, during the birth of a daughter, she had seen herself, the physician and the baby from an elevated point of view. She felt pity for the crying baby and returned. Since this event had occurred outside the scope of the study, it was not taken into consideration.

Two further (male) patients (№ 18 and 88, with no detailed recollections) 'fell from the trainer', case **P** (№ 111) literally into the arms of medical staff at a Reha center. Resuscitation efforts started immediately, but apparently they took a while. From the patient's point of view the experience felt like switching channels on a TV set. At first he felt a weakness when coming down from heavy labor, but suddenly the trainer was gone and he found himself standing in a corridor, with human-like silhouettes, tall ones and small ones, against a bright light. He wondered how he did get there and where the bicycle had gone. He felt uneasy and that he did not belong there. It was no agreeable state. When he opened his eyes, he saw 4-5 heads above him and felt recurrent pressure on his chest. But once more the scenery changed back to the shadows against the light. Pressure on his chest continued and started to hurt. He noticed a mask on his face and could hear his own gasps through this mask. He heard excited voices and someone said: "He is here now. Oxygen!" Clinical staff confirmed that two reanimations had been necessary. Treatment was continued at the General Hospital. The patient assures that his impressions didn't feel like a dream. They rather felt like two alternative realities. The spectacular impression resulted in just moderate Greyson points: 1 (8) + 1 (13) + 1 (16) = **3**.

Case **Q** (№ 115), a vital female 52 years old on the day of her CA, just wanted to dance at the 'Africa Days' (Donauinsel, Vienna). Acute shoulder pain interfered with her plans. She looked for help at the orthopedic department of the General Hospital, and there she almost booked a still more exclusive event. During the 2nd infiltration she passed out. She watched herself from behind, in T-shirt and jeans, standing in front of a big wooden door. The door was right before her face and remained closed. **Q** supposes that she went through this experience shortly before waking up. It might have been a dream. Greyson points: 1 (5) + 1 (16) = **2**.

Case **R** (№ 122), a male 76 years old when the CA occurred, collapsed while walking his dog (near the apartment). Neither he suffered a blackout nor a 'white-out' (like case **D**); according to his account, he didn't collapse at all. He remembers the walk with his dog and was convinced that also for the next 14 days or so he continued his sojourn in a green environment (he is biologist and forester). The prolonged and undeniable presence of medical staff led him to speculate on a pavilion of the General Hospital in the Vienna outskirts. To his opinion, all these people were his guests and he as their host was responsible for their well-being. He observed these duties with great pleasure and diligence, he cooked for them and he sang for them (Rock'n'Roll), although lying in bed all the time. After some days this bizarre behavior caused more amusement than concern among staff and relatives, since it slowly gave way to returning insight and reason. Greyson point **1** (1).

Three days before publication of the notorious ‘Ibiza video’, case **S** (№ 124), a female 39 years old when her CA did happen, made her rounds as security in the Chancellor’s Office (an innocent coincidence). After 2 floors she had difficulties to breathe. She fainted in the ambulance. Her reanimation took 48 min. She remembers a nightmare featuring ‘bone men’ that threatened death to her with bones in their hands. She was horrified by the suspicion that these aggressors moved closer to her bed each time she closed her eyes. Therefore, she desperately sought to keep her eyes open. She is convinced that for her this was a matter of life and death. In her case, no agreeable or peaceful state did unfold. She didn’t enjoy talking about it. In this group with detailed recollections, she was the other (besides case **C**) with a Greyson score zero.

Case **T** (№ 125). The highest Greyson score of all 126 patients was reached in the very last interview of this study. **T** (a female 46 years old at the time of the CA 195 d before the interview) suffered her CA on a Saturday morning at home and was reanimated by her partner within short delay. In the interview she stated with simple words that she had been “on the other side”, in the “afterlife”. It was very beautiful there, and the experience didn’t feel like a dream. She floated towards a stairway in the middle of lots of sky (“viel Himmel”). At the top of the stairs she saw her father (deceased 27 years ago). Also other people were around, all in a good mood and standing together in groups, men and women. She began to float upstairs to join her father, but he sent her back. Her time had not yet come. She had to return to her place to finish her mission. **T** is a catholic and indicated to frequent ceremonies only once a year. Before this event, she never heard about NDEs. Greyson points: 1 (1) + 2 (5) + 2 (6) + 2 (7) + 1 (8) + 2 (9) + 2 (11) + 2 (13) + 2 (15) + 2 (16) = **18**.
